# Plant Protein-Derived Bioactive Peptides: From Mechanistic Promise to Health-Promoting Functional Foods

**DOI:** 10.3390/nu18172826

**Published:** 2026-08-28

**Authors:** Maria Czernicka, Patrycja Sowa-Borowiec, Anna Wondołowska-Grabowska

**Affiliations:** 1 Department of Bioenergetics, Food Analysis and Microbiology, Faculty of Technology and Life Sciences, University of Rzeszów, Zelwerowicza 8b St., 35-601 Rzeszow, Poland; 2 Department of General and Inorganic Chemistry, Faculty of Chemical Engineering and Technology, Cracow University of Technology, 31-155 Crakow, Poland; patrycja.sowa-borowiec@pk.edu.pl; 3 Institute of Agroecology and Plant Production, University of Environmental and Life Sciences, Grunwaldzki Sq. 24A, 50-363 Wrocław, Poland; anna.wondolowska-grabowska@upwr.edu.pl

**Keywords:** plant proteins, bioactive peptides, functional foods, bioavailability, food matrix, sensory quality, translational barriers

## Abstract

Plant protein-derived bioactive peptides have attracted increasing interest as potential ingredients for health-promoting functional foods because of their reported cardiometabolic, antioxidant, anti-inflammatory, immunomodulatory, antimicrobial, gastrointestinal, and satiety-related activities. However, the field remains dominated by peptide discovery, in silico prediction, enzyme-inhibition assays, simulated digestion, and preclinical models, whereas successful translation into clinically supported and technologically viable food products is still limited. This review critically examines the gap between mechanistic promise and functional food implementation. It integrates evidence on plant protein sources, peptide-generation strategies, structure–activity relationships, gastrointestinal stability, intestinal transport, local gut activity, food-matrix interactions, processing effects, encapsulation, sensory constraints, human efficacy, regulatory substantiation, commercial feasibility, and consumer acceptance. Particular attention is given to the limited predictive value of isolated in vitro activity when peptides are exposed to digestion, epithelial barriers, complex food matrices, realistic processing conditions, and achievable dietary doses. The review also highlights that systemic absorption is not the only relevant pathway, as selected peptides may act locally within the gastrointestinal tract. Overall, the evidence indicates that peptide discovery should not be treated as the principal endpoint of research. Future progress will require translation-oriented development in which bioactivity, digestion stability, matrix compatibility, sensory quality, manufacturing reproducibility, realistic intake, human evidence, and regulatory credibility are evaluated as interdependent criteria. The most promising plant-derived peptides will therefore be those that retain sufficient activity and acceptability under real conditions of food production and consumption.

## 1. Introduction

Plant proteins are increasingly positioned at the intersection of food-system sustainability, nutritional adequacy, and health-oriented product innovation. Rebalancing protein supply towards plant sources may reduce several environmental pressures associated with food production, although the magnitude of this benefit depends on crop choice, agricultural practice, fractionation intensity, processing requirements, and the nutritional and sensory quality of the final food [1,2]. At the same time, plant protein ingredients are no longer considered solely as substitutes for animal proteins. Their structural diversity, technological functionality, and occurrence in legumes, cereals, pseudocereals, oilseeds, and protein-rich processing side streams make them versatile substrates for value-added food design and circular resource use [3,4,5]. One particularly active research direction concerns peptide sequences encrypted within parent proteins that may be released by gastrointestinal digestion, controlled enzymatic hydrolysis, germination, non-thermal pretreatment, or microbial fermentation [6,7,8,9,10,11].

Plant protein-derived bioactive peptides are generally short amino-acid sequences whose activity depends not only on amino-acid composition and primary sequence but also on molecular size, charge, hydrophobicity, conformation, terminal residues, and accessibility within the precursor protein [12,13,14]. The terminology, nevertheless, requires precision. A sequence predicted by database mining, molecular docking, quantitative structure–activity modelling, or machine-learning tools should be regarded as a candidate peptide until its identity and activity have been experimentally confirmed [15,16]. Likewise, activity in a chemical, enzyme-inhibition, or cell-based assay does not by itself establish physiological efficacy, because the peptide may be degraded, transformed, poorly absorbed, or inactive within the target food matrix [17,18,19,20]. With this distinction in mind, plant-derived peptides and peptide-rich hydrolysates have shown antihypertensive, antioxidant, anti-inflammatory, antidiabetic, hypolipidaemic, antimicrobial, gut-barrier-modulating, and satiety-related potential through targets that include angiotensin-converting enzyme, renin, dipeptidyl peptidase IV, carbohydrate-hydrolysing enzymes, redox-sensitive signalling pathways, inflammatory mediators, lipid-metabolism regulators, and intestinal nutrient-sensing pathways [21,22,23,24,25,26].

Despite this expanding mechanistic literature, the evidence base remains strongly weighted towards in silico predictions, purified-enzyme assays, simulated digestion studies, and short-term cell or animal models, whereas robust human evidence is limited and unevenly distributed across peptide classes and health outcomes [15,24,27,28]. This imbalance is particularly important because bioactivity is highly context-dependent. A peptide identified in a purified hydrolysate may be transformed or destroyed during food processing, bind to proteins, polyphenols, lipids, carbohydrates, minerals, or phytates within the matrix, and undergo further cleavage by gastric, pancreatic, and brush-border peptidases [29,30,31,32]. Peptides that survive digestion must then either act locally in the gastrointestinal tract or cross the intestinal epithelium through size- and sequence-dependent pathways before systemic effects become plausible [33,34,35]. Standardised digestion procedures such as INFOGEST have improved comparability across studies, but simulated digestion remains an approximation of human physiology and cannot, on its own, demonstrate absorption or clinical efficacy [20,36]. Caco-2 monolayers and human-induced pluripotent stem cell-derived intestinal epithelial models add information on epithelial transport and metabolism, yet they still simplify mucus, immune, microbial, vascular, and interindividual determinants of exposure [35,37].

The principal limitation of the field is therefore no longer peptide discovery alone but the preservation and demonstration of function under realistic conditions of consumption. Food-matrix composition, pH, ionic strength, heat treatment, fermentation, storage, and processing intensity can all modify peptide stability, solubility, release, and biological activity [27,38,39,40,41]. Formulation strategies, including encapsulation, spray-drying, controlled-release systems, and biopolymer-based carriers, may improve peptide stability and mask undesirable sensory properties, but their effectiveness is matrix-specific and may introduce new constraints related to release kinetics, cost, scale-up, regulatory status, and consumer perception [19,34,42,43]. Sensory quality is equally consequential. Hydrophobic peptide sequences and high degrees of hydrolysis frequently generate bitterness, astringency, beany notes, or lingering off-flavours, meaning that a technologically stable and biologically active ingredient may still fail at the level of acceptable food intake [44,45,46,47]. Finally, regulatory substantiation requires defined active constituents, realistic intake assumptions, safety assessment, and well-designed human studies capable of supporting a cause-and-effect relationship with a beneficial physiological outcome [48,49,50].

Recent reviews have comprehensively described plant peptide sources, production and purification strategies, biological activities, bioavailability, and potential applications [1,7,8,9,19]. More recent syntheses have also begun to consider translational barriers and differences in the level of experimental validation [51]. However, much of this literature remains organised primarily around peptide sources, production technologies, or individual activity classes. The distinctive focus of the present review is therefore not the expansion of the catalogue of potentially bioactive peptides but the end-to-end evaluation of their functional-food translation. Specifically, we examine how gastrointestinal transformation, local or systemic biological exposure, food-matrix and processing interactions, formulation and sensory constraints, achievable dietary dose, human efficacy, manufacturing reproducibility, regulatory substantiation, commercial feasibility, and consumer acceptance interact to determine whether mechanistic promise can be retained under realistic conditions of food production and consumption.

**Figure 1:**
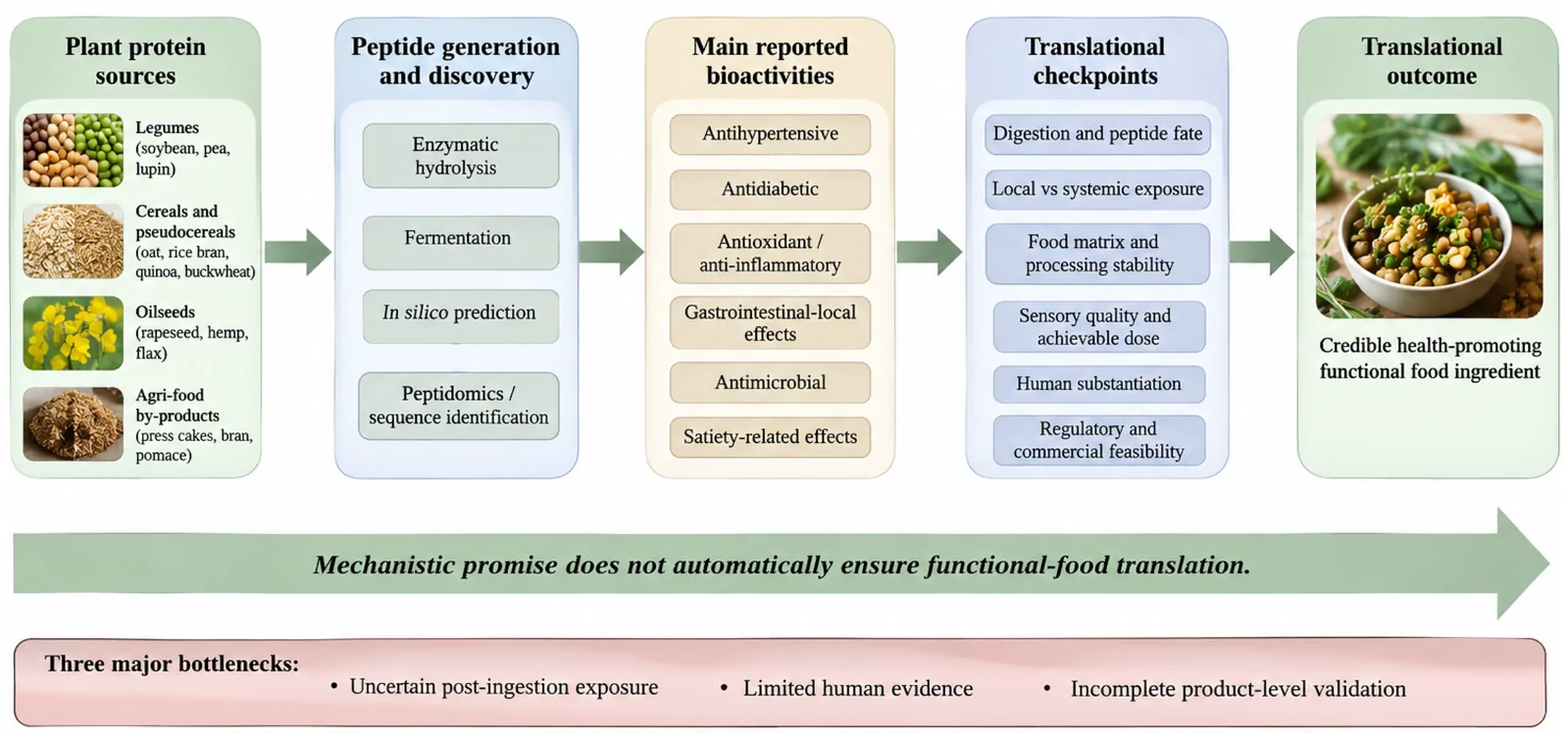
Translational framework for plant protein-derived bioactive peptides. The scheme links plant sources and reported bioactivities with the key biological, technological, clinical, and regulatory checkpoints that determine functional-food translation. Major bottlenecks include uncertain post-ingestion exposure, limited human evidence, and incomplete product-level validation.

Accordingly, this review addresses three explicit questions: (i) How far does the available evidence for plant protein-derived bioactive peptides progress from computational prediction and isolated in vitro activity towards digestion-aware, in vivo, and human substantiation? (ii) At which translational checkpoints–including gastrointestinal stability, local or systemic exposure, food-matrix incorporation, processing, formulation, sensory performance, and dose feasibility–does the plausibility of promising peptide candidates most commonly diminish? (iii) What combination of biological evidence, product-level validation, manufacturing reproducibility, regulatory substantiation, and consumer acceptability is required for a plant-derived peptide or peptide-rich hydrolysate to progress from mechanistic promise to a credible health-promoting functional food ingredient? By addressing these questions within a common translational framework, this review seeks to distinguish peptide discovery from evidence sufficient to support realistic functional-food development and to identify the points at which future research can most effectively close the gap between the two. Figure 1 summarises the translational framework adopted in this review, linking plant peptide sources and reported bioactivities with the key biological, technological, clinical, and regulatory checkpoints that determine functional-food readiness.

## 2. Literature Search and Selection Strategy

This review was conducted as a structured critical narrative review rather than a systematic review or meta-analysis. Its purpose was not to provide an exhaustive quantitative synthesis of all published studies but to critically integrate heterogeneous evidence relevant to the translation of plant protein-derived bioactive peptides from discovery and mechanistic validation to functional food applications. The review framework therefore encompassed peptide sources and generation strategies, structure–activity relationships, reported biological activities, gastrointestinal stability, intestinal transport and local gastrointestinal effects, food-matrix and processing interactions, delivery systems, sensory constraints, human evidence, regulatory substantiation, commercial feasibility, and consumer acceptance.

Relevant literature was identified through structured searches of PubMed, Scopus, and Web of Science, supplemented by targeted searches in ScienceDirect, SpringerLink, Wiley Online Library, MDPI, and Google Scholar. Search terms were used individually and in combination and included “plant protein-derived peptides”, “bioactive peptides”, “plant peptides”, “protein hydrolysates”, “legume peptides”, “cereal peptides”, “oilseed peptides”, “ACE-inhibitory peptides”, “DPP-IV inhibitory peptides”, “antioxidant peptides”, “anti-inflammatory peptides”, “antimicrobial peptides”, “satiety peptides”, “gastrointestinal digestion”, “bioavailability”, “intestinal transport”, “Caco-2”, “INFOGEST”, “food matrix”, “encapsulation”, “sensory properties”, “functional foods”, and “health claims”. Reference lists of relevant reviews and primary studies were also examined to identify additional publications directly related to the translational questions addressed in this review.

Publications were selected according to their relevance to one or more predefined domains of the review. Priority was given to peer-reviewed primary studies reporting peptide identification or hydrolysate characterisation, experimentally validated biological activity, simulated gastrointestinal digestion, intestinal transport or bioavailability, food-matrix and processing effects, sensory performance, animal studies, and human intervention trials. Recent reviews and systematic reviews were used primarily to provide broader context, identify methodological trends, and support areas in which primary evidence was highly dispersed. Seminal studies were retained where they established important methodological or mechanistic concepts, while official regulatory documents were included when required to evaluate health-claim substantiation, novel-food considerations, or market-entry requirements. Studies focused exclusively on non-plant peptides were generally outside the scope of the review, except where they provided a relevant methodological or clinical benchmark for evaluating the maturity of plant-derived peptide evidence.

Because the evidence base spans fundamentally different experimental levels, findings were interpreted according to their translational proximity to human food use rather than treated as equivalent. In silico predictions and molecular docking were considered hypothesis-generating; biochemical and enzyme-inhibition assays were regarded as evidence of potential activity; cell-based, simulated-digestion, and intestinal-transport studies provided progressively stronger information on biological plausibility and exposure; animal studies supported in vivo plausibility; and controlled human interventions were assigned the greatest relevance for substantiating physiological effects. Particular weight was given to studies that combined peptide identification with post-digestion characterisation, realistic food matrices, exposure assessment, or clinically relevant outcomes.

No formal risk-of-bias tool, quantitative study-quality score, or PRISMA-based study-selection procedure was applied because of the broad narrative scope and substantial methodological heterogeneity of the included evidence, which ranged from computational prediction and biochemical assays to food-processing studies, animal experiments, clinical trials, and regulatory documents. This represents a limitation of the review and means that selection and interpretation may be subject to narrative-review bias. To reduce this limitation, conclusions were formulated in relation to the underlying level of evidence, with particular caution applied to findings derived exclusively from in silico, chemical, or isolated in vitro models and greater inferential weight assigned to primary studies incorporating digestion, biological exposure, realistic food matrices, and human outcomes.

## 3. Plant Protein Sources for Bioactive Peptide Release

### 3.1. Legumes and Pulses

Legumes are the dominant matrix in plant peptide research because of their high protein content, favourable amino-acid profile, and abundance of storage proteins such as vicilin, legumin, conglutin, glycinin, and β-conglycinin, which are susceptible to controlled proteolysis [33,52]. Soybean remains the most extensively studied source, providing the canonical anti-inflammatory peptide lunasin as well as hydrolysates with ACE- and DPP-IV-inhibitory activities [23,53,54,55]. Pea has emerged as a parallel platform for cardiometabolic peptide research, partly because of its growing technological and commercial relevance in plant-protein ingredients [47,55].

Beyond soybean and pea, lentil, chickpea, faba bean, mung bean, cowpea, lupin, and peanut have yielded multifunctional peptides with antihypertensive, antioxidant, antidiabetic, anti-inflammatory, and immunomodulatory potential [56,57]. Faba bean has been re-evaluated as an underused temperate-climate protein source whose hydrolysates and gastrointestinal digestates show competitive antihypertensive, antioxidant, and anti-inflammatory profiles [58,59]. Germination followed by simulated digestion has generated sequence-confirmed antihypertensive and antioxidant peptides from lentil and faba bean [60,61], while chickpea hydrolysates have demonstrated blood-pressure-lowering effects in spontaneously hypertensive rats and immunomodulatory effects in human macrophage models [62,63]. Lupin conglutins have also been explored through in silico digestion as sources of potentially bioavailable dual ACE-I/DPP-IV inhibitory peptides [64]. Comparative peptidomics across legume genera is now enabling a more quantitative ranking of sources by peptide yield, sequence profile, and predicted or validated bioactivity [65,66,67].

### 3.2. Cereals and Pseudocereals

Cereal grains and their bran fractions remain underexploited as sources of bioactive peptides despite the global availability of wheat, rice, oat, maize, and sorghum. Wheat bran and gluten hydrolysates have demonstrated ACE-inhibitory, antioxidant, and immunomodulatory activities, including effects in human peripheral blood mononuclear cell models [68,69,70]. Oat protein provides an example of an integrated in silico-to-experimental workflow, with LC-Q-TOF-MS identification of the multifunctional peptide VW showing DPP-IV-, ACE-, and antioxidant-related activity [71]. Rice bran is particularly notable because the Leu-Arg-Ala peptide is among the few plant-derived peptides evaluated in a double-blind, randomised, placebo-controlled human trial, where it showed antihypertensive effects [72]. Pseudocereals, including quinoa, amaranth, and buckwheat, are attractive because their albumins and globulins differ from those of true cereals and may generate distinct peptide profiles [5,73]. Quinoa hydrolysates have yielded peptides with ACE-, DPP-IV-, and cholesterol-esterase-inhibitory potential [71,74], while buckwheat- and hemp-rich meals have been associated with favourable post-prandial satiety hormone responses in healthy volunteers [75]. These findings support the view that cereal, pseudocereal, and pulse staples remain underused relative to their potential as sources of health-relevant peptides [76,77].

### 3.3. Oilseeds

Oilseeds offer a particularly attractive translational opportunity because defatted meals from rapeseed/canola, flaxseed, sesame, sunflower, hemp, chia, and other oil crops are abundant by-products of oil extraction and are often underused in human food applications [3,78]. Comparative hydrolysis studies of flaxseed, rapeseed, sunflower, sesame, and soybean proteins have validated in silico activity predictions and shown that oilseed hydrolysates can exhibit ACE-inhibitory activity comparable to that reported for some dairy-derived peptide systems [79]. Rapeseed napin and cruciferin have yielded novel ACE-inhibitory peptides confirmed by molecular docking, including FQW, FRW, and CPF [80].

Among oilseed sources, flaxseed and chia are especially relevant to the translational theme of this review. Flaxseed-derived peptides are unusual in that calmodulin-dependent phosphodiesterase inhibition and serum detectability have been assessed directly in vivo [81,82]. Chia protein hydrolysates have yielded bioavailable peptidomes with antioxidant and ACE-inhibitory potential confirmed using Caco-2 transwell models [83]. Walnut meal peptides have also shown in vivo hypolipidaemic activity in high-fat-diet rat models, providing a mechanistic benchmark for lipid-lowering plant peptides [84].

### 3.4. Agro-Industrial By-Products and Emerging Sources

Bioactive peptide research is increasingly aligned with by-product valorisation and circular food-system strategies. Oilseed meals, brewer’s spent grain, rice bran, pulse fractionation residues, fruit seeds, and bread waste have all been explored as low-cost, low-footprint sources of peptide-rich hydrolysates [4,85]. These substrates are particularly relevant for functional food development because they combine nutritional value, sustainability, and potential technological functionality. Emerging and underutilised sources further broaden the plant peptide landscape. Millet, teff, sorghum, amaranth, and other minor grains may provide alternative peptide profiles, while aquatic plants such as duckweed have recently attracted attention through machine-learning-guided discovery of dual ACE/DPP-IV inhibitory peptides from Lemnaceae hydrolysates [5,16]. Specialty botanicals, including maca, turmeric, and *Zizyphus jujuba*, have also yielded immunomodulatory, antimicrobial, or orally relevant peptide candidates [18,86,87]. However, for many of these sources, evidence remains mainly at the discovery or preclinical stage, and their relevance for functional foods will depend on scalability, sensory performance, safety, and clinical substantiation. Thus, peptide yield and predicted activity alone are insufficient criteria for source selection; the resulting ingredient must also be reproducible, process-compatible, sensorially manageable, and suitable for realistic food applications.

## 4. Health-Promoting Potential

**Figure 2:**
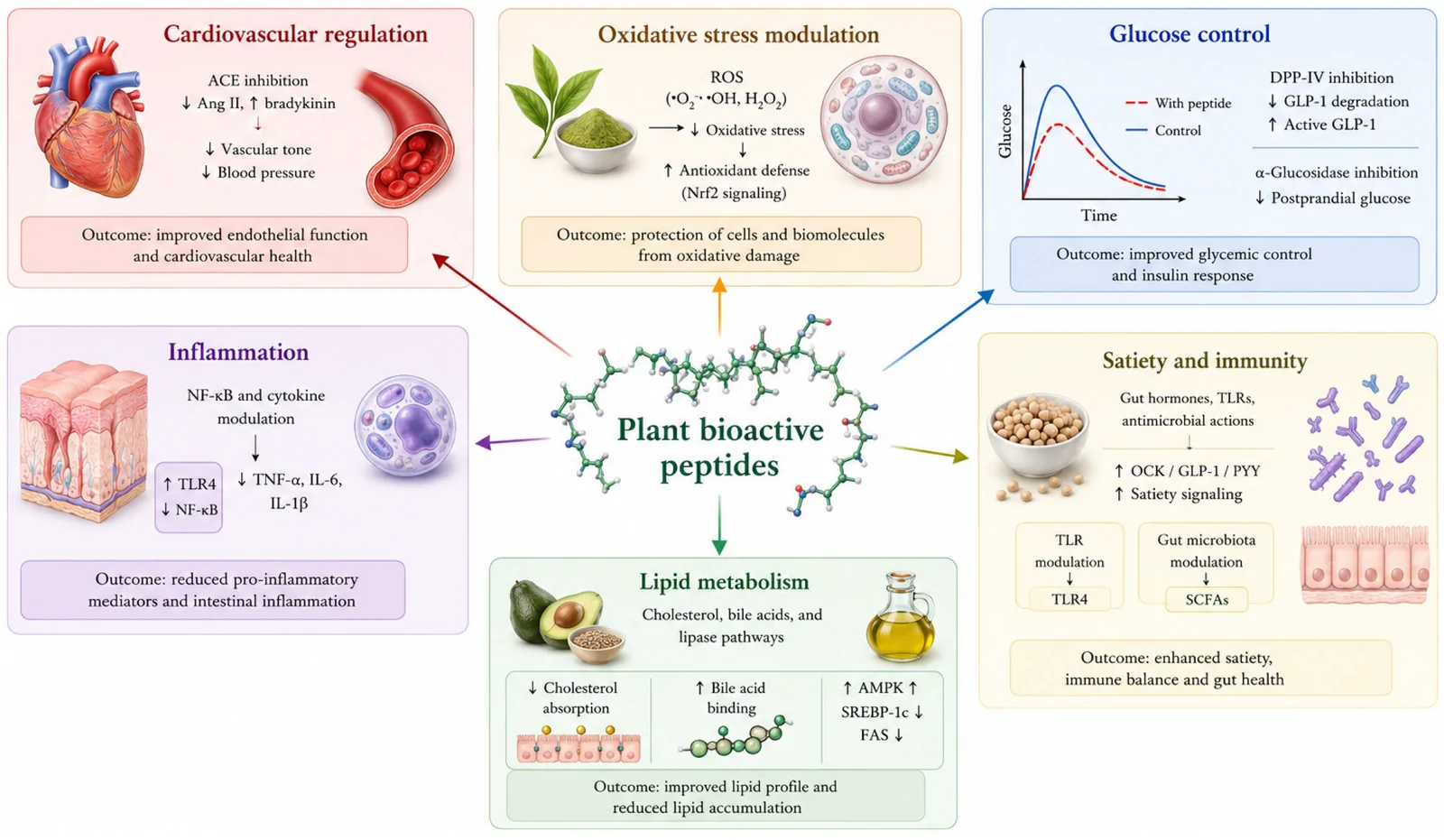
Health-promoting mechanisms proposed for plant-derived bioactive peptides. The scheme summarises major mechanistic axes, including cardiovascular regulation, oxidative-stress modulation, glucose control, lipid metabolism, inflammation, satiety, immunity, antimicrobial activity, and gut-barrier support. Most proposed mechanisms are supported primarily by in vitro, in silico, and animal evidence, whereas clinical substantiation remains limited and uneven across peptide sources and health outcomes.

The health-promoting potential of plant protein-derived bioactive peptides spans cardiometabolic, antioxidant, anti-inflammatory, immunomodulatory, antimicrobial, gastrointestinal, and satiety-related axes [1,8,25,26]. These activities are mechanistically plausible because short peptide sequences can interact with enzymes, receptors, redox-sensitive signalling systems, inflammatory mediators, lipid-metabolism pathways, and intestinal barrier or microbiota-related targets [12,23,33,88]. Figure 2 summarises the principal health-promoting mechanisms proposed for plant-derived bioactive peptides, while also indicating that most mechanistic evidence remains derived from in vitro and animal studies rather than from consistent clinical substantiation. Cardiometabolic mechanisms are currently the most extensively documented, partly because their molecular and physiological readouts, including ACE and renin inhibition, DPP-IV and α-glucosidase inhibition, cholesterol esterase and lipase modulation, plasma lipid changes, and blood pressure reduction, are experimentally tractable in vitro and in vivo [21,24,55,71]. By contrast, immune, microbiota-related, and satiety effects often involve more complex and context-dependent pathways [22,76,89]. Therefore, the biological activities discussed below should be interpreted as proposed or partially validated mechanisms rather than uniformly established clinical effects [15,27,28]. Their translational value, therefore, depends not only on mechanistic plausibility but also on whether these effects persist after digestion, matrix incorporation, processing, and consumption at feasible doses.

### 4.1. Cardiometabolic Effects

Cardiometabolic effects constitute the most extensively investigated area of plant protein-derived bioactive peptide research. This prominence reflects the availability of experimentally tractable molecular targets, including angiotensin-converting enzyme (ACE), renin, dipeptidyl peptidase-IV (DPP-IV), α-glucosidase, α-amylase, pancreatic lipase, cholesterol esterase, and selected regulators of hepatic lipid and glucose metabolism. Among these, ACE inhibition remains the dominant model, partly because it provides a clear biochemical link between peptide structure and blood-pressure regulation [21,24,55,71].

ACE catalyses the conversion of angiotensin I into the vasoconstrictor angiotensin II and contributes to the degradation of bradykinin, a vasodilatory peptide. Plant-derived ACE-inhibitory peptides have been identified in soybean, pea, lentil, chickpea, faba bean, mung bean, lupin, wheat, oat, rice bran, quinoa, rapeseed, flaxseed, chia, hemp, duckweed, and other protein sources [16,21,24,55,60,65,70,71,80,83]. Structure–activity analyses generally indicate that short sequences containing hydrophobic, aromatic, or proline residues, particularly at the C-terminal region, are favoured for ACE inhibition because they can interact with the catalytic site and stabilise peptide–enzyme binding [14,21,90]. Renin inhibition, which acts upstream of ACE in the renin–angiotensin–aldosterone system, represents an additional antihypertensive mechanism, although it remains less frequently investigated for plant peptides [24,30,91]. Evidence from spontaneously hypertensive rat models supports the blood-pressure-lowering potential of selected plant peptide fractions, including low-molecular-weight wheat bran, pea, and chickpea hydrolysates [62,70]. However, animal models remain mechanistic benchmarks rather than substitutes for human substantiation. The rice bran Leu-Arg-Ala peptide trial is therefore particularly important because it remains one of the few double-blind, randomised, placebo-controlled human studies involving a plant-derived peptide and showing a modest but significant antihypertensive effect [72]. Overall, antihypertensive peptides illustrate both the maturity of the discovery field and the scarcity of clinically validated plant peptide ingredients.

Beyond blood-pressure regulation, plant-derived peptides have been investigated for antidiabetic effects, particularly through DPP-IV inhibition and inhibition of carbohydrate-hydrolysing enzymes. DPP-IV degrades incretin hormones such as GLP-1 and GIP, which contribute to insulin secretion and post-prandial glucose regulation. Peptides capable of inhibiting DPP-IV may therefore prolong endogenous incretin activity and support glycaemic control [23,55,71]. Multifunctional peptides showing both ACE- and DPP-IV-inhibitory activities are common in soybean, pea, lupin, and oat hydrolysates, suggesting that cardiometabolic activities frequently overlap at the peptide-sequence level [55,64,66,71]. α-Glucosidase and α-amylase inhibition provide complementary mechanisms by delaying carbohydrate digestion and glucose release, although most evidence for these effects remains based on in vitro assays and preclinical models.

Plant peptides may also influence lipid metabolism and hepatic lipid handling. Proposed mechanisms include inhibition of pancreatic lipase or cholesterol esterase, reduced cholesterol micellar solubilisation, bile-acid binding, modulation of HMG-CoA reductase, activation of AMPK-related pathways, stimulation of hepatic fatty-acid oxidation, and regulation of LDL receptor expression [23,84,92,93,94]. Walnut meal peptides, for example, have been shown to improve lipid profiles and reduce hepatic lipid accumulation in high-fat-diet animal models [84]. Similarly, fermented legume systems and plant hydrolysates have been associated with lipid-lowering and hepatoprotective effects, although the contribution of specific peptide sequences is often difficult to separate from the broader food matrix, fermentation-derived metabolites, fibre, phenolics, and other bioactive constituents [68,94].

Taken together, cardiometabolic effects represent the strongest and most coherent area of plant peptide research, but they also expose the central translational limitation of the field. ACE, DPP-IV, α-glucosidase, and lipid-metabolism assays provide useful mechanistic screening tools, yet their results cannot be directly extrapolated to clinical benefit. Functional food relevance requires evidence that peptide-rich ingredients remain active after digestion and processing, are delivered at realistic intake levels, and produce measurable physiological effects in humans. Thus, cardiometabolic peptides are promising not because their in vitro activity is abundant but because this area provides the clearest framework for moving from mechanistic screening towards clinically substantiated food applications.

### 4.2. Antioxidant, Anti-Inflammatory, and Immunomodulatory Effects

Antioxidant, anti-inflammatory, and immunomodulatory effects represent a second major activity cluster of plant protein-derived bioactive peptides, particularly in the context of oxidative-stress-related damage, chronic low-grade inflammation, metabolic dysfunction, and gastrointestinal immune homeostasis [12,26,27,54,57,88]. These mechanisms are biologically interconnected because reactive oxygen species, redox-sensitive signalling, inflammatory transcription factors, cytokine networks, and immune-cell activation frequently reinforce one another in chronic cardiometabolic and intestinal disorders [27,63,68]. Plant-derived peptides have therefore been investigated not only as direct radical scavengers or metal chelators but also as modulators of endogenous antioxidant defence, NF-κB and MAPK signalling, nitric oxide and prostaglandin E_2_ production, macrophage activation, and Th1/Th2/Th17-related immune polarisation [12,53,86,88].

However, the level of evidence remains highly dependent on the experimental model. Antioxidant activity is still frequently reported using chemical assays such as DPPH, ABTS, FRAP, ORAC, reducing power, and metal-chelation tests, whereas anti-inflammatory and immunomodulatory effects are usually evaluated in macrophage, epithelial, or peripheral blood mononuclear cell models [12,26,63,68]. These approaches are useful for screening and mechanism generation, but they do not establish physiological efficacy after oral intake and cannot fully reproduce digestion, absorption, intracellular uptake, metabolism, endogenous antioxidant defence, food-matrix interactions, or clinically relevant biomarkers [15,27,28]. At the mechanistic level, plant peptides may act as hydrogen or electron donors, chelate transition metals, reduce lipid peroxidation, or interact with cellular antioxidant pathways such as the Nrf2/Keap1/ARE axis [12,26,57]. Peptides containing Tyr, Trp, His, Cys, and Met are frequently associated with antioxidant potential because these residues can stabilise radicals, participate in electron transfer, or interact with reactive oxygen species [12,65,77,79]. Antioxidant peptides have been reported from legumes, cereals, pseudocereals, and oilseeds, including phenolic-rich legume genera, wheat bran, quinoa, buckwheat, flaxseed, rapeseed, sunflower, sesame, and soybean hydrolysates [65,77,79].

Interpretation of antioxidant peptide activity requires particular caution because plant matrices often contain phenolic compounds, Maillard reaction products, residual proteins, lipids, minerals, and other redox-active constituents that may contribute to measured antioxidant capacity. In phenolic-rich legumes, comparative work suggests that peptides can contribute substantially to antioxidant activity, but separation of peptide-specific effects from matrix-derived antioxidant effects remains essential [65]. Therefore, antioxidant claims should ideally be supported by peptide identification, fractionation, sequence confirmation, post-digestion testing, and cellular assays that assess oxidative-stress protection under biologically relevant conditions. Without this level of characterisation, antioxidant activity remains a useful screening endpoint but a weak predictor of physiological efficacy.

Cross-level consistency has nevertheless been demonstrated in selected studies. Xu et al. [95] showed that the <5 kDa soybean meal hydrolysate fraction displaying the strongest DPPH-scavenging and reducing activity also increased hepatic SOD and GSH-Px activities and decreased MDA levels after oral administration in mice. Importantly, the same size-defined fraction was carried from chemical screening into in vivo antioxidant assessment, although its complex peptide composition still precluded attribution of the effect to individual sequences.

Anti-inflammatory and immunomodulatory activities are mechanistically more complex than direct antioxidant effects. Plant peptides and hydrolysates have been reported to modulate cytokine balance, NF-κB and MAPK signalling, nitric oxide and prostaglandin E_2_ production, macrophage activation, lymphocyte responses, and Th1/Th2/Th17-related immune polarisation [27,54,63,68,86,88]. Such effects are relevant to chronic low-grade inflammation, metabolic syndrome, intestinal inflammation, and immune homeostasis. However, immune-related endpoints are strongly context-dependent and often differ according to cell type, inflammatory stimulus, peptide dose, digestion status, and the complexity of the food matrix.

Soybean lunasin remains one of the best-known plant peptides with reported anti-inflammatory, immunomodulatory, epigenetic, and chemopreventive relevance [53]. Other plant protein sources have also provided promising evidence. Wheat gluten hydrolysates have demonstrated modulation of cytokine responses in human peripheral blood mononuclear cells, including effects on Th1/Th17-related inflammatory signalling [68]. Chickpea protein hydrolysates, alone or in combination with vitamin D, have shown immunomodulatory effects in macrophage-based models [63]. Maca protein hydrolysate-derived peptides have also been investigated for immunomodulatory activity, supporting the broader relevance of botanical protein sources beyond conventional legumes and cereals [86].

Human evidence remains limited but instructive. In the Lupine-1 trial, 33 healthy adults completed a 28-day open-label intervention in which they consumed a beverage providing 1 g day^−1^ of *Lupinus angustifolius* protein hydrolysates. The intervention was associated with reduced production of selected pro-inflammatory Th1 cytokines in stimulated peripheral blood mononuclear cells, increased cellular antioxidant capacity, and a reduction in the LDL-C/HDL-C ratio, without major safety concerns [96]. However, the absence of a placebo-controlled comparator, the short intervention period, and the healthy study population limit causal and clinical interpretation. Moreover, peptide-specific systemic exposure was not assessed, preventing a direct pharmacokinetic–pharmacodynamic link between individual peptide sequences and the observed responses. The study therefore represents an important step beyond isolated in vitro activity while also illustrating the substantial evidence gap between a peptide-rich hydrolysate producing measurable biological responses and a clinically substantiated peptide-specific effect.

Overall, antioxidant, anti-inflammatory, and immunomodulatory effects are highly relevant to the health-promoting potential of plant protein-derived peptides, but their translational status is less mature than that of selected cardiometabolic endpoints. Chemical antioxidant assays and inflammatory cell models are valuable for screening and mechanism generation, yet they cannot by themselves support functional food claims. Future studies should integrate digestion-stable peptide identification, cellular oxidative-stress and immune models, gut-barrier systems, and human biomarkers of inflammation or redox status. This would help distinguish true peptide-mediated effects from broader matrix effects and clarify whether these mechanisms can be preserved under realistic conditions of food processing and consumption.

### 4.3. Gut-Related, Antimicrobial, and Satiety-Modulating Effects

Gut-related, antimicrobial, and satiety-modulating effects represent an emerging but less clinically substantiated area of plant protein-derived bioactive peptide research. Importantly, the gastrointestinal tract may serve not only as a barrier to peptide absorption but also as a biologically relevant site of action. Peptides released during digestion, fermentation, or controlled hydrolysis may interact locally with brush-border enzymes, epithelial cells, tight-junction complexes, mucosal immune pathways, enteroendocrine signalling systems, or luminal microorganisms, meaning that low systemic recovery should not automatically be interpreted as biological inactivity [22,33,89]. A peptide-specific example is the rapeseed napin-derived dipeptide Thr–Leu, which in a Caco-2/RAW264.7 coculture model underwent epithelial transport predominantly through PepT1 while simultaneously improving barrier-related expression of occludin and ZO-1 and reducing inflammatory mediators. This type of model is particularly informative because it links peptide identity with epithelial exposure and a defined local biological response, although the relatively high experimental peptide concentration and in vitro setting still limit direct extrapolation to dietary efficacy [97].

Microbiota-related effects require even greater interpretive caution because direct sequence-specific evidence remains scarce. In an irinotecan-induced intestinal-injury model, a soybean peptide preparation improved intestinal architecture, increased occludin and ZO-1 expression, reduced inflammatory cytokines, and partially restored microbial diversity, including increases in Lactobacillus and Bifidobacterium [98]. Similarly, a walnut peptide preparation alleviated DSS-induced colitis in mice while improving barrier integrity and altering the relative abundance of several bacterial taxa [99]. However, both studies evaluated complex peptide preparations rather than single sequence-confirmed peptides, so the observed microbiota shifts cannot be attributed causally to individual peptide sequences or assumed to mediate the host response. Counter-evidence further supports this caution: Li et al. [100] showed that duodenal administration of soy protein hydrolysate in growing pigs increased microbial richness but reduced several butyrate- and propionate-producing taxa, increased potentially pathogenic and protein-fermenting bacteria, and was accompanied by higher colonic pro-inflammatory cytokines. Thus, microbiome responses to plant protein hydrolysates appear to be source-, dose-, host-, and context-dependent. Microbiota modulation should therefore be regarded as peptide-specific only when the active sequences are sufficiently characterised and microbial changes are linked to functional metabolites or mechanistic host outcomes rather than inferred from compositional shifts alone.

Antimicrobial activity represents a distinct translational pathway because plant-derived peptides may act either as health-related gastrointestinal modulators or as functional agents for controlling foodborne microorganisms. Their proposed mechanisms include membrane permeabilisation, disruption of membrane potential, interaction with intracellular targets, and interference with microbial metabolism; however, these effects depend strongly on peptide charge, hydrophobicity, amphipathicity, length, and the surrounding physicochemical environment [27,101,102]. Sequence- or fraction-specific primary studies provide encouraging examples. Roytrakul et al. [87] identified a turmeric-derived peptide fraction with antibacterial and antioxidant activity, illustrating the potential of botanical proteins beyond conventional food crops. More directly relevant to food preservation, Santangelo et al. [85] showed that antifungal peptides recovered from bread waste retained activity after freeze-drying when protected with selected polysaccharides, demonstrating that antimicrobial efficacy can be strongly influenced by formulation and processing. These findings also expose a key translational limitation: antimicrobial activity measured in purified systems cannot be assumed to persist in foods, where ionic strength, pH, lipids, proteins, polysaccharides, and proteolytic enzymes may alter peptide–microbe interactions. Consequently, antimicrobial peptides intended for food applications should be evaluated in the actual target matrix and under realistic storage conditions, with efficacy interpreted together with stability, sensory compatibility, safety, and intended regulatory function.

Satiety-related effects are mechanistically plausible but remain difficult to attribute to individual peptide sequences. Food-derived peptides may influence appetite regulation through enteroendocrine pathways involving cholecystokinin, GLP-1, and peptide YY, as well as through nutrient-sensing receptors and gut–brain signalling [22,89]. Rubisco-derived peptides provide peptide-level evidence that specific plant sequences can interact with appetite-related signalling pathways, supporting a direct mechanistic basis for local gastrointestinal action [89]. However, the human evidence is much less peptide-specific. Neacsu et al. [75] showed that meals containing hemp and buckwheat proteins altered post-prandial gastrointestinal hormones, insulin responses, and plasma amino-acid profiles in healthy volunteers, but these effects cannot be assigned specifically to bioactive peptides because the interventions involved whole protein-rich meals rather than sequence-defined peptide preparations. This distinction is critical for translation: changes in satiety hormones after consumption of plant proteins or hydrolysates may reflect amino-acid release, gastric emptying, matrix effects, or broader nutrient signalling rather than a direct action of individual peptides. Future studies should therefore combine characterised peptide or hydrolysate interventions with realistic food formats, defined intake levels, validated appetite outcomes, and, where possible, biomarkers linking peptide exposure to enteroendocrine responses.

Overall, gut-related, antimicrobial, and satiety-modulating effects broaden the relevance of plant-derived peptides beyond mechanisms that require systemic exposure. However, the strength of evidence varies considerably across these outcomes. Local epithelial effects can increasingly be linked to defined peptide sequences, whereas microbiota and satiety responses are still often inferred from complex hydrolysates, whole proteins, or food matrices. Future progress will therefore depend on distinguishing peptide-specific mechanisms from broader matrix effects and on validating local gastrointestinal activity using digestion-aware models, realistic exposure conditions, and clinically relevant endpoints.

## 5. From Peptide Activity to Functional Foods

The transition from plant protein-derived peptide activity to a functional food product requires more than the identification of bioactive sequences. A peptide that inhibits an enzyme in vitro or shows activity in a cell model must remain sufficiently stable during food processing, gastrointestinal digestion, and storage; reach its intended site of action; retain activity in a complex food matrix; be acceptable sensorially; and ultimately demonstrate efficacy at realistic intake levels [19,27,32,34]. Bioavailability, matrix compatibility, formulation, sensory quality, clinical evidence, regulatory substantiation, and consumer acceptance, therefore, represent interdependent checkpoints in the development of peptide-enriched functional foods.

### 5.1. Bioavailability, Intestinal Transport, and Digestion Stability

Bioavailability remains one of the most critical bottlenecks in the translation of plant protein-derived bioactive peptides into functional foods. Although numerous peptides exhibit strong activity in vitro, only a fraction of ingested sequences can be expected to survive gastrointestinal digestion, cross the intestinal barrier, and reach systemic circulation in an active form. Oral delivery exposes peptides to a cascade of biological barriers, including gastric acid, pepsin, pancreatic proteases, brush-border peptidases, the mucus layer, epithelial tight junctions, efflux transporters, and hepatic first-pass metabolism [5,19,32,34,103]. Consequently, in vitro bioactivity should be regarded as a preliminary indicator rather than proof of physiological efficacy.

Standardised digestion models are increasingly used to assess peptide bioaccessibility. The INFOGEST protocol provides a harmonised static approach for simulating oral, gastric, and small-intestinal digestion under physiologically relevant conditions of pH, enzyme activity, electrolyte composition, and digestion time. In the INFOGEST 2.0 workflow, samples sequentially undergo an oral phase (pH 7.0, 2 min), a gastric phase with pepsin (pH 3.0, 2 h), and an intestinal phase with pancreatin and bile salts (pH 7.0, 2 h), all at 37 °C [104,105]. However, the standard protocol does not reproduce brush-border peptidase activity, epithelial transport, hepatic first-pass metabolism, microbiota-mediated transformations, or interindividual variability [103,106]. Therefore, post-digestion bioactivity assays should ideally be complemented by intestinal permeability models and targeted peptide identification to distinguish peptides that are merely bioaccessible from those with realistic potential for absorption and systemic activity [32,103].

Caco-2 monolayers remain the most widely used cellular model for intestinal permeability because they differentiate into enterocyte-like cells with tight junctions, microvilli, and selected transport systems. Apparent permeability coefficients are useful for ranking peptide transport potential, although they cannot fully reproduce the complexity of the human intestinal epithelium. Caco-2 monolayer Papp values for ACE-inhibitory peptides typically fall in the 10^−8^ cm/s range, and even comparative work using human-induced pluripotent stem cell-derived intestinal epithelial cells has produced only modestly higher values, underscoring the limits of any single permeability surrogate [35,37]. Human-induced pluripotent stem cell-derived intestinal epithelial cells may offer a more physiologically relevant alternative for evaluating peptide transport and intestinal metabolism, whereas passive diffusion models such as PAMPA can support preliminary screening but lack active transport, metabolism, and cell-based barrier properties [35,107,108,109,110].

At the mechanistic level, the proton-coupled peptide transporter PepT1 (SLC15A1) is the principal carrier involved in the uptake of di- and tripeptides. However, its capacity for longer bioactive sequences is limited [35]. Larger peptides may cross the epithelium through transcytosis or paracellular diffusion, but these routes are generally inefficient and highly structure-dependent [32,34]. These constraints explain why peptide length, hydrophobicity, charge, proline content, secondary structure, and resistance to proteolysis are increasingly considered essential design parameters for functional food applications [13,14,30,103].

Direct serum measurements support a sobering picture. In vivo oral gavage of six flaxseed-derived peptides yielded only two detectable peptides in rat serum, highlighting the low systemic availability of many food-derived sequences [82]. Similarly, in vivo gastrointestinal tracking of *Zizyphus jujuba* peptides revealed major discrepancies between in silico predictions and physiologically present sequences after digestion [18]. Systematic analyses of structural determinants of peptide stability point to length, hydrophobicity, Pro/Hyp content, and secondary structure as primary protectants against proteolysis [13,20,36]. The implication for functional food design is therefore clear: in vitro bioactivity is necessary for candidate selection, but it is profoundly insufficient as a predictor of in vivo efficacy.

Importantly, systemic absorption is not the only plausible route of action. Some peptides may act locally within the gastrointestinal tract by interacting with epithelial cells, immune mediators, intestinal enzymes, microbial communities, or gut-barrier-related pathways. This possibility broadens the translational relevance of peptides with low systemic permeability, but it also increases the need for models that distinguish bioaccessibility, local intestinal activity, epithelial exposure, and true systemic bioavailability.

### 5.2. Food Matrix Effects and Processing Stability

The food matrix is rarely a neutral carrier of bioactive peptides. Rather, it represents a complex physicochemical environment that can modulate peptide release, stability, solubility, sensory perception, gastrointestinal accessibility, and biological activity. Polyphenols, starches, lipids, dietary fibre, divalent cations, minerals, phytates, and residual proteins can either protect peptides from degradation or bind and inactivate them, illustrating the “two faces of the same coin” paradox described for peptide–matrix interactions [19,27,29,30]. This dual role is particularly relevant for plant-derived peptides, which are often delivered in protein-rich, fibre-rich, phenolic-rich, mineral-rich, or fermented food systems rather than in purified aqueous solutions.

Protein–peptide and peptide–peptide interactions are among the most immediate matrix effects. Residual proteins and released peptides may associate through hydrophobic interactions, hydrogen bonding, electrostatic forces, disulfide exchange, and aggregation phenomena, thereby altering solubility, digestibility, and bioaccessibility [29]. In legume systems, phytate–protein–calcium ternary complexes may further reduce protein digestibility and mineral bioavailability, indicating that anti-nutritional interactions should be considered when designing peptide-enriched foods from pulses and legume protein isolates [31]. These effects are important because a hydrolysate that shows strong activity in a buffer may behave differently when incorporated into a dense plant-based matrix containing fibre, phenolics, minerals, and residual intact proteins.

Carbohydrates and reducing sugars can also modify peptide behaviour. Maillard reactions between peptide amino groups and reducing sugars may alter peptide structure, antioxidant potential, colour, flavour, digestibility, and biological activity. Uncontrolled glycation may reduce bioactivity, promote aggregation, or generate undesirable off-flavours, whereas controlled Maillard-type interactions have been explored as a strategy to improve antioxidant capacity and mask bitterness [27,111]. This illustrates an important formulation principle: the same chemical interaction may be either detrimental or useful depending on reaction conditions, target activity, and the intended food format.

Lipid–peptide interactions may influence peptide release and bioavailability through hydrophobic complex formation, interfacial adsorption, or incorporation into emulsified systems [29]. Emulsified lipids can delay gastric emptying and may protect selected peptides against rapid proteolysis, but lipid oxidation products may also react with nucleophilic peptide residues and reduce biological activity [27]. Similarly, polysaccharides such as pectin, alginate, guar gum, and gum arabic may form electrostatic complexes with cationic peptides, modifying solubility, viscosity, release behaviour, intestinal residence time, and interaction with taste receptors [29,30]. These interactions are central to functional food design because they determine whether a peptide is released at the appropriate stage of digestion or remains trapped within the matrix.

Processing conditions further determine whether a peptide-enriched food retains its intended activity. Moderate heat treatment can denature proteins, inactivate protease inhibitors, increase enzyme accessibility, and improve peptide release, whereas excessive heating, baking, extrusion, prolonged storage, or repeated thermal exposure can promote aggregation, Maillard reactions, peptide degradation, oxidation, and loss of activity [27,40,41]. In legumes, pressure cooking, microwaving, and conventional boiling have been shown to differentially affect protein digestibility and antioxidant peptide release, confirming that processing effects are matrix- and method-dependent rather than universally beneficial or detrimental [38].

Product-format studies further illustrate the translational importance of matrix effects. Incorporation of plant protein hydrolysates into pectin-based soft confections altered colour, texture, sensory acceptance, and bioactivity retention, with outcomes varying substantially according to processing conditions and formulation parameters [39]. Similarly, faba bean, soybean, and pea digestates exhibit species- and fraction-dependent bioactivities, indicating that functional claims cannot be generalised across plant protein sources without considering cultivar, fractionation, protein composition, matrix structure, and digestion behaviour [2,58].

Non-thermal technologies, including high-pressure processing, pulsed electric fields, ultrasound, and related emerging approaches, may improve enzyme accessibility and peptide release while limiting thermal degradation [7,112]. However, their effects remain matrix-specific and require optimisation for each food system. For example, structural unfolding that facilitates enzymatic hydrolysis may also expose hydrophobic residues that intensify bitterness or promote aggregation. Therefore, processing should not be evaluated solely by peptide yield or in vitro bioactivity, but also by digestion stability, sensory quality, scalability, and compatibility with the intended food matrix.

Overall, food matrix and processing effects represent a central translational checkpoint between peptide discovery and functional food development. A candidate peptide or hydrolysate should therefore be tested not only in purified form but also in the actual or intended product matrix after realistic processing, storage, and digestion. This formulation-aware approach is essential for determining whether the biological potential observed in vitro can be preserved under conditions of real consumption.

### 5.3. Encapsulation and Delivery Systems

Encapsulation and delivery-system design have become major strategies for improving the technological and biological performance of bioactive peptides in functional foods. Their principal aims are to protect peptides during processing, storage, and gastrointestinal transit; reduce premature degradation; modulate release kinetics; improve compatibility with food matrices; and mask undesirable sensory attributes such as bitterness or astringency [19,42,111]. In this context, encapsulation should be viewed not only as a protection strategy but also as a formulation tool that links peptide stability, bioaccessibility, sensory quality, and product feasibility.

A wide range of delivery systems has been explored for food-derived peptides, including liposomes, solid lipid nanoparticles, nanostructured lipid carriers, protein-based nanoparticles, carbohydrate-based hydrogels, coacervates, spray-dried microcapsules, electrospun fibres, emulsions, and Pickering systems [32,34,43,113]. For functional food applications, carbohydrate- and protein-based carriers are particularly relevant because of their compatibility with conventional food matrices and their established use in food processing. Alginate, chitosan, pectin, starch, gum arabic, zein, soy proteins, and whey proteins can form particles, gels, or coacervates that modulate peptide release and reduce direct interaction with taste receptors [112,113]. Such systems may therefore address both bioavailability and palatability, although their performance remains highly dependent on carrier composition, peptide properties, pH, ionic strength, and the target food matrix. Spray-drying is especially attractive from an industrial perspective because it is scalable, relatively cost-effective, and compatible with powdered ingredients, bakery applications, beverages, and dry mixes. Spray-drying of wheat gluten hydrolysate with maltodextrin and potato starch carriers preserved antioxidant activity and modulated peptide release under simulated gastrointestinal digestion [69]. Similarly, spray-dried peptide systems have been incorporated into bakery products with partial retention of bioactivity and bitterness masking [40,41]. Freeze-drying and polysaccharide-based wall materials have also been used to protect bread-waste-derived peptides with antifungal activity, although such systems may expose trade-offs between processing stability, cost, and bioactivity retention [85].

Lipid-based systems, including liposomes, solid lipid nanoparticles, nanostructured lipid carriers, and emulsions, may protect hydrophobic or amphiphilic peptides and alter their interaction with digestive fluids and epithelial surfaces [32,34,43]. Pickering emulsions and biopolymer-stabilised systems are particularly relevant for food applications because they can be integrated into emulsion-based products while limiting direct peptide exposure to taste receptors and digestive enzymes. However, lipid-based carriers also introduce potential constraints related to oxidative stability, ingredient labelling, manufacturing cost, release control, and compatibility with low-fat or minimally processed product formats.

Despite these advantages, encapsulation should not be presented as a universal solution to the translational limitations of plant protein-derived bioactive peptides. Encapsulation efficiency, peptide retention, and in vitro protection are not equivalent to improved oral bioavailability, local intestinal activity, or clinical efficacy [20,42]. A system that protects a peptide during simulated digestion may also delay or prevent release at the intended site of action. Conversely, a carrier that improves release may expose the peptide to proteolysis, matrix binding, or sensory detection. Therefore, release kinetics should be evaluated together with digestion stability, epithelial exposure, food-matrix behaviour, and the intended physiological target. Several additional barriers are important for functional food translation. Wall materials, particle size, processing conditions, storage stability, regulatory status, scale-up feasibility, cost, and consumer perception of micro- or nanoencapsulated ingredients can all influence whether an encapsulated peptide system is commercially viable [32,34]. These issues are particularly relevant for plant peptide ingredients intended for everyday foods rather than pharmaceutical-like supplements. From a formulation perspective, the most promising delivery systems are therefore those designed together with the target food matrix, expected processing route, sensory profile, and intended site of action. Encapsulation should be integrated early into peptide development, rather than used as a final corrective step after bioactivity, bitterness, or instability problems have already emerged.

### 5.4. Sensory Barriers and Mitigation Strategies

Sensory acceptability is a central translational barrier for plant protein-derived bioactive peptides because biologically active hydrolysates must be consumed at realistic and repeated intake levels to become functional food ingredients. Bitterness is the most consistent limitation, particularly in hydrolysates enriched in low-molecular-weight and hydrophobic peptides. This creates a “flavour–function duality”: the same structural features that may favour enzyme inhibition, membrane interaction, or antioxidant activity can also promote activation of bitter taste receptors and reduce consumer acceptance [44,45].

Beaniness and astringency add further constraints in legume-based systems, especially pea, soy, and faba bean ingredients. Fermentation with selected lactic acid bacteria can improve the sensory profile of pea protein-based beverages, but it does not necessarily eliminate off-notes [47]. Moreover, fermentation and enzymatic pretreatments used to maximise peptide release may themselves generate bitter non-volatile compounds, sour notes, or interfere with the structural properties required in products such as extruded meat analogues [114]. Thus, sensory quality is not independent of peptide generation; it is shaped by the same processing decisions that determine bioactivity. Several mitigation strategies have been proposed, including controlled hydrolysis, exopeptidase treatment, flavour masking, cyclodextrin complexation, adsorption, encapsulation, and microbial fermentation [32,45,46]. However, these approaches should be evaluated critically. Debittering may increase process complexity, cost, and formulation constraints, and some methods may remove or modify the same hydrophobic peptide fractions responsible for the desired biological activity. Therefore, sensory mitigation should be integrated with peptidomic profiling and bioactivity testing rather than applied as a final corrective step.

Consumer-facing evidence reinforces this point. Health or environmental labelling may improve expectations and willingness to try plant-based products, but it cannot fully compensate for poor taste, unpleasant mouthfeel, or persistent off-flavours. Intrinsic sensory quality remains a major determinant of acceptability and willingness to pay [115]. For plant peptide-enriched foods, this means that biological activity, sustainability, and technological feasibility will not be sufficient unless the final product is sensorially acceptable at the intended dose.

### 5.5. Clinical Evidence and Substantiation

The clinical evidence base for plant protein-derived bioactive peptides remains limited relative to the volume of in silico, in vitro, and preclinical research, but it is more heterogeneous than suggested by a focus on isolated examples. Human intervention studies have evaluated peptide-rich or peptide-enriched preparations derived from pea, soybean, rice bran, lupin, hemp, and other plant proteins, with outcomes including blood pressure, glycaemic control, body composition, oxidative and inflammatory markers, and muscle-protein metabolism. However, these studies differ substantially in intervention characterisation, sample size, duration, comparator, dose, and clinical endpoint, and most have tested complex hydrolysates or peptide mixtures rather than purified sequence-defined peptides. Human evidence should therefore be interpreted according to both study design and the degree to which the administered peptide material was characterised and linked to the observed physiological response. Table 1 summarises the human intervention studies selected for their translational relevance in this review.

Taken together, the human studies summarised in Table 1 show that plant-derived peptide preparations have progressed beyond preclinical testing in several domains, particularly blood-pressure regulation, but the clinical evidence remains fragmented. Most interventions involved peptide-rich hydrolysates or peptide networks rather than purified, sequence-defined peptides, making causal attribution to individual bioactive sequences difficult. Sample sizes were frequently small, intervention periods were generally short, and direct assessment of peptide exposure was uncommon. Moreover, positive findings were not universal: the controlled lunasin trial did not demonstrate significant improvement in the principal cardiometabolic outcomes, while in the hemp study the contribution of the added peptide fraction could not be fully separated from that of the parent protein. Cardiovascular outcomes currently provide the most developed human evidence, whereas glycaemic, immune, and satiety-related effects remain supported mainly by pilot or short-term studies. Thus, the principal clinical gap is not the complete absence of human experimentation but the scarcity of independently replicated trials that combine well-characterised peptide preparations, realistic intake, clinically meaningful endpoints, and evidence linking exposure to physiological response.

Future clinical studies should prioritise well-characterised, reproducible peptide preparations tested at realistic intake levels with appropriate comparators and clinically relevant outcomes. Where feasible, peptide exposure or biomarker assessment should link the administered preparation to the observed physiological response, while complex hydrolysates should be analytically characterised for composition and batch reproducibility. Without this integration, positive human findings remain insufficient for peptide-specific causal attribution and robust functional-food claim substantiation [24,48,103].

### 5.6. Regulatory, Commercialisation and Consumer-Acceptance Barriers

Regulatory substantiation represents a final and often decisive barrier between mechanistic promise and functional food implementation. In the European Union, peptide-related health claims fall under Regulation (EC) No. 1924/2006 and require clear characterisation of the active food constituent, evidence of a cause-and-effect relationship with a beneficial physiological effect, appropriate dose justification, and support from well-designed human studies [48,50,122]. This evidentiary bar is particularly difficult for complex plant protein hydrolysates because their peptide profiles may vary with cultivar, growing conditions, protein extraction, hydrolysis parameters, processing, storage, and the final food matrix. Most innovative bioactive-compound claims, including those related to hydrolysates and peptide-containing ingredients, have struggled because of insufficient human efficacy data, incomplete constituent characterisation, or inability to establish causality [50,123].

The European regulatory context also requires attention to novel food considerations when peptide isolates, engineered hydrolysates, new production processes, or unconventional plant sources are used. In such cases, toxicological data, allergenicity assessment, intake estimates, compositional characterisation, and manufacturing reproducibility may be required before market entry [124,125]. The soy protein–LDL precedent illustrates both the scale of human evidence required and the difficulty of translating mechanistic plausibility into an authorised claim [122,126]. Even in fermented foods, where bioactive peptides are biologically plausible mediators, current systematic evidence remains insufficient to support new EFSA-authorised claims [127].

In the United States, regulatory routes include GRAS status for food ingredients, authorised health claims requiring Significant Scientific Agreement, qualified health claims with lower evidentiary certainty, and structure–function claims for conventional foods or dietary supplements [48,128]. In practice, many peptide-containing products are more likely to rely on general structure–function wording than on disease-risk reduction or authorised health claims. This pathway may facilitate market entry, but it provides weaker scientific differentiation and may not support the level of credibility required for clinically positioned functional foods.

Japan and China provide additional regulatory models relevant to plant peptide-based functional foods. In Japan, health-related food claims may be communicated through the Foods for Specified Health Uses (FOSHU) or Foods with Function Claims (FFC) systems. FOSHU requires individual governmental evaluation and authorisation, whereas FFC operates through pre-market notification, with responsibility for scientific substantiation and safety resting primarily with the business operator [129]. In China, health foods are subject to registration or filing depending on product and ingredient status; products using ingredients outside the Health Food Raw Materials Catalogue and most first-imported health foods require registration, while permitted health functions are linked to the official function catalogue [130]. Novel peptide isolates or unconventional plant sources may additionally require safety assessment as new food raw materials before food use [131]. These frameworks further emphasise the need to align peptide characterisation, safety, manufacturing reproducibility, and evidence of function with the intended regulatory pathway early in product development.

Commercialisation is constrained not only by regulation but also by scalability, production time, cost, ingredient standardisation, and product reproducibility. Industrial production requires consistent plant protein isolates or concentrates, optimised hydrolysis or fermentation conditions, and reliable downstream processing. Although enzymatic hydrolysis can efficiently release bioactive peptides, commercial proteases and process control increase production costs, while fermentation may require longer processing times and careful control of strain performance and product consistency [6,56]. Downstream fractionation can become an even greater economic bottleneck: ultrafiltration is comparatively scalable, whereas repeated chromatographic purification substantially increases processing time, solvent use, product losses, and cost, particularly when highly purified individual peptides are targeted [132]. Consequently, peptide-rich hydrolysates or standardised fractions may be more commercially realistic for functional foods than highly purified single peptides when sufficient biological activity can be retained. Batch-to-batch variability in plant protein composition further complicates standardisation [2].

Consumer acceptance adds a further translational filter. Koning et al. [133] showed that acceptance of alternative protein products is shaped by sensory quality, food neophobia, expected nutritional value, health and sustainability framing, price, trust, and willingness to pay. Amayo et al. [115] further demonstrated that although health- or environment-related labelling may increase expected liking and willingness to try plant-based products, intrinsic sensory quality remains a major determinant of repeat purchase and willingness to pay. For plant peptide-enriched foods, this means that regulatory credibility, clinical evidence, and sustainability narratives are unlikely to compensate for poor taste, inconsistent texture, or unclear consumer communication.

These constraints indicate that regulatory and commercial strategy should be built into peptide development from the beginning. Analytical characterisation, intake assumptions, safety, allergenicity, digestion stability, food-matrix compatibility, clinical endpoints, sensory feasibility, consumer-relevant product formats, and claim wording should be considered early, rather than after a bioactive sequence has already been identified.

## 6. Translational Barriers and Future Priorities

The evidence reviewed above indicates that the principal weakness of the field is not a shortage of candidate peptides but an imbalance between discovery and translational validation. Computational and biochemical screening have advanced rapidly, whereas evidence on gastrointestinal persistence, biologically relevant exposure, performance in realistic food matrices, clinically meaningful efficacy, and product-level reproducibility remains comparatively sparse. Consequently, translational readiness should not be judged by mechanistic potency alone but by the extent to which evidence is retained across successive biological, technological, clinical, and regulatory checkpoints. This distinction provides the basis for identifying the barriers that currently most strongly limit functional-food translation and for defining the priorities required to overcome them.

At the earliest stage of the translational continuum, in silico screening, peptidomics, molecular docking, and machine-learning approaches can efficiently generate and prioritise candidate sequences for experimental validation [7,16,64]. Cournoyer et al. [16], for example, demonstrated the value of this approach by combining machine-learning-guided discovery with experimental validation of multifunctional peptides from duckweed hydrolysates. However, even experimentally confirmed activity at this stage remains insufficient to establish translational readiness because it does not demonstrate gastrointestinal persistence, biologically relevant exposure, activity within a realistic food matrix, achievable effective dose, or human efficacy [15,18]. Discovery-stage evidence should therefore be interpreted as hypothesis-generating and candidate-prioritising rather than as evidence of functional-food effectiveness.

To avoid treating heterogeneous evidence as equivalent, the literature reviewed here can be organised into a translational evidence hierarchy (Table 2). This framework is not intended as a formal risk-of-bias instrument or quantitative study-quality score; rather, it defines the inferential contribution of different evidence types, the features that strengthen their translational relevance, and the additional validation required before stronger conclusions are justified. Importantly, the hierarchy should not be interpreted as a strictly linear development pathway. Gastrointestinal exposure, product-level feasibility, and biological efficacy may need to be evaluated iteratively, and strength at one level cannot compensate for failure at another. Translational confidence, therefore, increases not simply with the number of studies available but with convergence between molecular characterisation, biologically relevant exposure, activity under realistic food conditions, in vivo plausibility, and controlled human evidence. This framework integrates methodological considerations related to peptide discovery, oral bioavailability, food-matrix effects, and functional-food substantiation [15,17,24,29,48,103].

Viewed through this translational hierarchy, three bottlenecks emerge as particularly consequential. First, biologically relevant exposure after ingestion remains uncertain. For peptides intended to exert systemic effects, this requires evidence of gastrointestinal persistence, epithelial passage, and exposure at concentrations compatible with the proposed mechanism; for peptides acting locally, persistence and activity within the intestinal lumen or at the epithelial interface may be sufficient but must be demonstrated directly rather than inferred from low systemic absorption [17,18,103]. Second, human substantiation remains disproportionately weak relative to the volume of computational, biochemical, and preclinical evidence. Controlled intervention studies using well-characterised plant peptide preparations at realistic intake levels are still uncommon, and positive physiological outcomes are rarely linked to confirmed peptide exposure or clearly defined active sequences [24,72]. Third, biological activity is frequently evaluated before product feasibility has been established. Food-matrix interactions, processing stability, achievable dose, sensory acceptability, and manufacturing reproducibility can each diminish the relevance of an otherwise promising peptide or hydrolysate [29,39,44]. These bottlenecks are interconnected rather than independent: failure to demonstrate exposure weakens causal interpretation, inadequate product-level validation undermines realistic dosing, and both ultimately reduce the likelihood that human efficacy and regulatory substantiation can be achieved.

At the downstream end of the translational continuum, regulatory and commercial readiness depends on convergence across the preceding evidence domains rather than on any single positive result. The peptide or peptide-rich ingredient, its manufacturing specification, biologically relevant dose, safety profile, clinical evidence, and proposed health positioning must refer to a sufficiently characterised and reproducible product [48,50,122]. This requirement is particularly challenging for complex plant hydrolysates, whose peptide composition may vary with cultivar, extraction, hydrolysis, processing, storage, and matrix incorporation. A positive mechanistic study or even an isolated favourable human outcome, therefore, cannot compensate for inadequate ingredient characterisation, uncertain dose–response relationships, or poor batch reproducibility. Commercial feasibility adds a further constraint because extensive purification, fractionation, analytical quality control, formulation, and clinical substantiation can substantially increase development complexity and cost, while consumer acceptance ultimately determines whether an efficacious ingredient can be incorporated into a product that is repeatedly consumed. Regulatory, commercial, and consumer barriers should therefore be viewed not as separate final obstacles, but as downstream tests of whether the biological and technological evidence has converged into a credible functional-food product.

These findings translate into three priority areas for the next stage of plant peptide research and development. First, researchers should move from activity-centred screening towards mechanism-specific translational validation. Candidate peptides should be sequence-confirmed and evaluated after gastrointestinal digestion because structural features strongly influence their persistence under digestive conditions [13,111]. Exposure should then be assessed according to the intended site of action: systemic mechanisms require evidence of intestinal passage and biologically relevant exposure, whereas locally acting peptides require direct demonstration of persistence and activity within the intestinal lumen or at the epithelial interface [17,103]. Standardised digestion approaches should increasingly be combined with targeted peptide identification and appropriate epithelial or local gastrointestinal models, while testing in realistic food matrices is needed to account for matrix-dependent changes in peptide availability [29]. Negative or null findings should also be reported because loss of activity during digestion, matrix incorporation, or transport is itself translationally informative.

Second, food-industry development should integrate biological activity with product feasibility from the earliest formulation stage. Plant-protein functionality and processing behaviour are strongly matrix-dependent, making early evaluation in the intended product format essential [2]. Peptide release, matrix compatibility, processing stability, achievable dose, and batch reproducibility should therefore be considered alongside biological activity rather than addressed sequentially. Product-format studies already show that incorporation of peptide-rich hydrolysates can alter texture, sensory acceptance, and retention of bioactivity [39]. Sensory quality requires particular attention because bitterness and other undesirable attributes are closely linked to peptide composition and hydrolysis conditions [46], while fermentation-based optimisation may improve but does not necessarily eliminate off-notes [47,114]. Manufacturing time, downstream processing requirements, and cost should likewise be incorporated into development decisions from the outset. Standardised peptide-rich preparations may therefore offer a more realistic route than highly purified individual peptides when comparable biological activity can be retained.

Third, clinical and regulatory development should begin with a sufficiently characterised and reproducible test material rather than with a loosely defined hydrolysate. Human interventions should use realistic intake levels, appropriate comparators, pre-specified clinically relevant outcomes, and adequate statistical power [24]. The rice-bran Leu–Arg–Ala study illustrates the type of controlled human evidence that remains uncommon for plant-derived peptides [72]. Regulatory substantiation additionally requires clear ingredient characterisation, dose justification, safety assessment, and evidence capable of supporting a cause-and-effect relationship [48,50]. These requirements should be considered before rather than after clinical testing, particularly because current EFSA guidance places substantial emphasis on characterisation of the food constituent and the relevance of the human evidence submitted [122]. Collectively, these priorities shift the field from maximising the number of newly identified peptide sequences towards selecting fewer candidates for which biological exposure, product feasibility, human efficacy, and regulatory credibility can be demonstrated as a coherent evidence chain.

## 7. Conclusions

This review extends previous source- and activity-centred syntheses by evaluating plant protein-derived bioactive peptides through an end-to-end translational framework that integrates peptide discovery with gastrointestinal fate, biologically relevant exposure, food-matrix performance, human substantiation, manufacturing reproducibility, and regulatory readiness. Across these levels, the evidence remains strongly weighted toward in silico, in vitro, and preclinical studies, whereas clinically substantiated benefits in humans are still comparatively scarce. Consequently, early-stage bioactivity should be interpreted as evidence of biological plausibility rather than as proof of functional-food efficacy.

Three priorities follow directly from this assessment. Researchers should prioritise sequence-confirmed candidates evaluated after gastrointestinal digestion and, where relevant, demonstrate biologically meaningful local or systemic exposure. Food developers should assess bioactivity together with matrix compatibility, sensory quality, realistic dose, scalability, and batch reproducibility from the earliest stages of formulation. Clinical developers should use well-characterised peptide preparations in appropriately designed human studies with relevant comparators and outcomes, while regulators should require evidence sufficient to support causal attribution and health-claim substantiation.

Ultimately, translational success should be judged not by maximal activity in isolated assays, but by the convergence of biological plausibility, digestion-aware exposure, product feasibility, human efficacy, and regulatory credibility. Plant-derived peptides are most likely to become meaningful functional-food ingredients when these dimensions can be demonstrated together under realistic conditions of production and consumption.

## Figures and Tables

**Table 1:** Selected human intervention studies evaluating plant protein-derived peptide preparations.

Plant Source/ Intervention	Study Design	Main Finding	Main Translational Limitation
Pea protein hydrolysate Li et al. [116]	Randomised, double-blind, placebo-controlled crossover; *n* = 7, 3 weeks	SBP decreased by approx. 5–6 mmHg compared with placebo	Very small sample; complex hydrolysate; no peptide- exposure assessment
Black soy peptides Kwak et al. [117]	Randomised, double-blind, placebo-controlled; 100 enrolled, 8 weeks; 4.5 g/day	Greater SBP reduction and improved oxidative-stress markers versus placebo	Peptide mixture; active sequences and systemic exposure not established
Rice-bran Leu–Arg–Ala (LRA) Ogawa et al. [72]	Randomised, double-blind, placebo-controlled; *n* = 100, 87 completed; 12 weeks; 43 μg LRA/day	Modest but significant reduction in SBP	Strongest sequence-linked example, but the effect was modest and requires independent replication
Lunasin-enriched soy extract Tabrizi et al. [118]	Triple-blind, placebo-controlled crossover; *n* = 31, 8 weeks/treatment	No significant improvement in major cardiometabolic risk factors	Important counter- evidence; enriched extract rather than purified peptide
Pea-derived peptide network NRT_N0G5IJ Chauhan et al. [119]	Randomised, double-blind, placebo-controlled pilot; *n* = 77 randomised, 12 weeks; 15 g/day	Small but significant reduction in HbA1c versus controls	Pilot study; modest effect; peptide network rather than single defined sequence
Lupin protein hydrolysate (Lupine-1) Cruz-Chamorro et al. [96]	Open-label intervention; *n* = 33, 28 days; 1 g/day	Changes in immune, antioxidant and lipid-related markers	No placebo comparator; short duration; peptide-specific exposure not established
Hemp protein + hemp- derived peptides Samsamikor et al. [120]	Randomised, double-blind crossover; *n* = 35; three 6-week treatments	HSP + peptides reduced 24 h BP versus casein	Hemp protein alone was also active; peptide-specific contribution remains difficult to isolate
Pea protein hydrolysates Gradl et al. [121]	Short-term randomised feeding study; *n* = 19 overweight men; 15 g PPH	Hydrolysate-dependent effects on energy intake, ghrelin, DPP-4 and gastric emptying	Small, male-only, acute study; no evidence of sustained satiety or weight-control benefit

Note: The table is not intended as an exhaustive census of all human studies but summarises interventions selected for their translational relevance to plant-derived bioactive peptides. Study designs, peptide characterisation, endpoints, and ability to attribute effects to specific peptide sequences vary substantially.

**Table 2:** Translational evidence hierarchy for plant protein-derived bioactive peptides.

Level	Typical Evidence	What It Supports	Main Limitation
**1. Discovery**	In silico prediction, docking, machine learning, peptidomics	Candidate prioritisation	Does not establish biological efficacy
**2. Mechanistic** **validation**	Enzyme assays, chemical assays, cell models	Biological plausibility and target interaction	Does not establish activity after oral intake
**3. Digestion** **and exposure**	INFOGEST, LC–MS/MS, epithelial/local GI models	Peptide persistence and local/systemic exposure	Does not establish physiological benefit
**4. Preclinical** **validation**	Oral animal studies, PK, physiological biomarkers	In vivo plausibility	Limited extrapolation to humans
**5. Product-level** **validation**	Real food matrix, processing, storage, sensory testing, realistic dose	Technological and formulation feasibility	Does not establish human efficacy
**6. Human** **substantiation**	Controlled human intervention studies	Physiological efficacy at realistic intake	Reproducibility and causal attribution may remain uncertain
**7. Translational** **readiness**	Safety, specifications, batch reproducibility, scale-up, regulatory and consumer evidence	Functional-food and market readiness	Requires convergence of preceding evidence

Note: The levels indicate increasing translational proximity rather than a formal risk-of-bias or study-quality score. Progression may be iterative rather than strictly linear.

## Data Availability

No new data were created or analyzed in this study. Data sharing is not applicable to this article, as this is a review based on previously published literature.
